# Effects of elastic band resistance training on lower limb strength and balance function in older adults: a systematic review and meta-analysis

**DOI:** 10.3389/fspor.2025.1649305

**Published:** 2025-11-06

**Authors:** Yunchen Meng, Yiling Hu, Wang Yang, Yaqi Xue, Sanjun Yang

**Affiliations:** 1 Department of Physical Education, China University of Mining and Technology-Beijing, Beijing, China; 2 Institute for Emergency Rescue Ergonomics and Protection, China University of Mining and Technology-Beijing, Beijing, China; 3 College of P.E and Sports, Beijing Normal University, Beijing, China

**Keywords:** elastic band, aging, lower limb strength, balance function, meta-analysis

## Abstract

**Introduction:**

This study systematically evaluated the effectiveness of elastic band resistance training (EBT) in improving lower limb strength and balance function in older adults with different health status.

**Methods:**

A comprehensive literature search was conducted across Scopus, EBSCO, Web of Science, PubMed, and Cochrane Library to identify randomized controlled trials (RCTs) published through December 30, 2024. Eligible studies included older adults in which the control group received either no intervention or basic treatment, and the experimental group received EBT in addition to the control protocol.

**Results:**

A total of 25 studies involving 1,318 participants were included. Meta-analysis demonstrated significant improvements in the EBT group compared with controls across multiple outcomes: leg extension test [SMD = 1.01, 95% CI (0.36, 1.66), *p* = 0.002], chair stand test [SMD = 2.04, 95% CI (0.60, 3.48), *p* = 0.006], timed up and go test [SMD = –1.41, 95% CI (–2.33, −0.49), *p* = 0.003] and functional reach test [SMD = 1.63, 95% CI (0.36, 2.90), *p* = 0.012].

**Conclusion:**

Elastic band resistance training may improve lower limb strength and balance function in older adults with different health conditions. Longer intervention durations may yield greater benefits in strength, while balance improvements can occur with shorter programs. Further high-quality studies are needed to confirm these effects.

**Systematic Review Registration:**

https://www.crd.york.ac.uk/PROSPERO/view/CRD420251021955, PROSPERO CRD420251021955.

## 1 Introduction

Maintaining physical function is essential for preserving independence in older adults. Diminished lower limb strength and impaired balance are major contributors to increased fall risk, which in turn raises healthcare costs and the likelihood of early institutionalization, ultimately compromising quality of life (1, 2). Balance is a fundamental component of daily functioning and relies on the integration of visual, vestibular, and proprioceptive inputs, in combination with muscular strength and reaction time. These systems work synergistically to coordinate movement through multisensory processing. However, with aging, balance function tends to decline due to progressive deterioration of both the central nervous system and neuromuscular pathways (3, 4). Skeletal muscle dysfunction, characterized by declines in strength, muscle atrophy, and reduced coordination, is a common consequence of aging and is widely recognized as an early indicator of balance impairment and increased fall risk (5–8). Early identification and intervention targeting muscle function are therefore essential components of fall prevention strategies.

Although combined strength and balance training has been shown to be effective in reducing fall incidence (9), traditional machine-based resistance training may pose adherence challenges for older adults, including elevated risk of injury and training dropout (10). In contrast, elastic resistance devices offer advantages such as affordability, portability, and ease of use. By utilizing lower resistance levels with higher repetition volumes, elastic resistance is considered a safe, accessible, and cost-effective approach to enhancing neuromuscular function, increasing muscle strength and power, and improving functional performance in older adults (10, 11).

Elastic band resistance training (EBT) has been demonstrated to improve gait, flexibility, and reduce fall risk in older adults, making it especially suitable for home-based exercise programs or as an adjunct to physical therapy interventions (12, 13). However, while numerous studies have reported the benefits of EBT, they often examine a broad spectrum of outcomes, including upper limb strength, endurance, flexibility, and body composition. This wide scope introduces potential heterogeneity and may reduce the certainty of evidence when evaluating its impact on specific core capacities such as lower limb strength and balance function (14–16). Consequently, there is a need for systematic and targeted evaluations focusing specifically on these two key outcomes. Clarifying the specific effects of EBT on lower limb strength and balance would enhance understanding of its role in maintaining functional ability and inform evidence-based fall prevention strategies in aging populations.

## 2 Methods

The systematic review and meta-analysis were conducted in accordance with the Preferred Reporting Items for Systematic Reviews and Meta-Analyses (PRISMA) guidelines (17, 18). The study protocol was retrospectively registered with PROSPERO (registration number: CRD420251021955).

### 2.1 Search strategy

A comprehensive literature search was conducted across five electronic databases: PubMed, Web of Science, Scopus, EBSCO, and the Cochrane Library. The search aimed to identify English-language studies published from database inception through December 30, 2024. The following Boolean search strategy was used: (older OR aging OR elderly OR advancing age OR advancing years) AND (variable resistance OR elastic band OR elastic tube OR rubber band OR Thera-band OR rubber tube OR elastic resistance) AND (strength OR balance OR flexibility OR agility OR mobility OR falls OR postural control OR motor control OR postural stability OR postural sway OR physical function).

### 2.2 Study selection criteria

Studies were eligible for inclusion in this review if they met the following criteria (a) The mean age of participants across the included studies was greater than 60 years, with no restrictions on demographic characteristics or medical conditions. (b) The intervention involved EBT as the primary method, without combination with other training modalities or equipment-based exercises. (c) The experimental group received EBT alone or in combination with basic treatments, while the control group received no intervention, only basic treatment, or the same co-interventions as the experimental group excluding EBT. (d) At least one of the following functional outcomes was assessed: leg extension test, chair stand test (CST), timed up and go test (TUG), or functional reach test (FRT). (e) Only randomized controlled trials (RCTs) published in peer-reviewed journals were included. Reviews, editorials, commentaries, and non–peer-reviewed publications were excluded. Studies were excluded if they met any of the following conditions (a) The intervention did not involve elastic band resistance training. (b) The outcome measures did not include assessments of lower limb muscle strength or balance function. (c) The full text of the study was unavailable through any means, or the reported data could not be extracted or utilized for analysis.

### 2.3 Data collection process and data items

The process of literature screening and data extraction was conducted independently by two researchers. Titles, abstracts, and full texts were screened according to predefined eligibility criteria. In cases of disagreement, a third researcher was consulted to reach a consensus. The following data was extracted from eligible studies: first author's name; publication year; study design; sample size; population demographic details including age, gender and health condition; intervention details and outcome measures.

### 2.4 Assessment of methodological quality

The same two reviewers used the Cochrane Risk of Bias tool to assess the risk of bias in the studies (17). The evaluation included the following domains: random sequence generation, allocation concealment, blinding of participants and personnel, blinding of outcome assessment, incomplete outcome data, selective reporting, and other potential sources of bias. Each study was judged as having a “low risk,” “high risk,” or “unclear risk” of bias. Any discrepancies were resolved through discussion or consultation with a third reviewer.

### 2.5 Statistical analysis

Meta-analyses were conducted using Stata 18 software. For each study, mean values and standard deviations of pre- and post-intervention changes were extracted. Standardized mean differences (SMD) with 95% confidence intervals (CI) were calculated to estimate pooled effect sizes. Heterogeneity was assessed using the *I*^2^ statistic. A fixed-effects model was applied when heterogeneity was low (*p* > 0.1, *I*^2^ < 50%); otherwise, a random-effects model was used. Sensitivity analyses were conducted to explore potential sources of heterogeneity. When substantial heterogeneity was identified, subgroup analyses were performed based on participants’ age, physical condition, and intervention duration. Publication bias was assessed through visual inspection of funnel plots and tested statistically using Egger's regression test. When publication bias was detected (*p* < 0.05), the trim-and-fill method was applied to adjust effect size estimates. Statistical significance was set at *p* < 0.05 for all analyses.

## 3 Results

### 3.1 Search results

**Figure 1:**
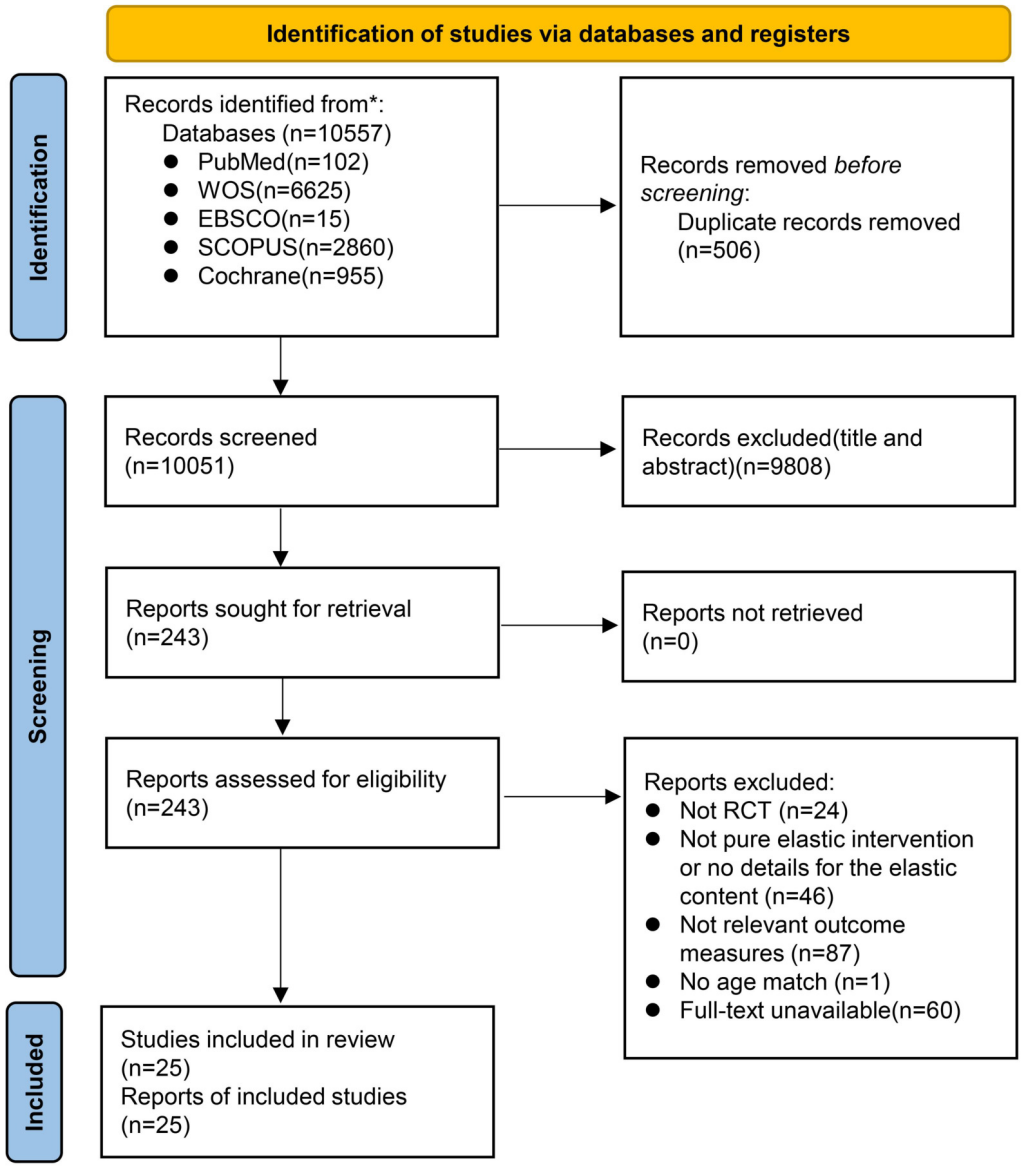
PRISMA flow diagram of the literature screening.

A total of 10,557 articles were initially identified through the database search. After screening titles and abstracts, 243 full-text articles were assessed for eligibility. Ultimately, 25 RCTs (12, 13, 19–41) were included in the meta-analysis, comprising 1,318 participants. The PRISMA flow diagram is presented in Figure 1.

Table 1 summarizes the characteristics of the included studies. Of the total participants, 712 were assigned to the EBT group and 606 to the control group. Seventeen studies (12, 13, 19, 20, 22–28, 33–36, 38, 40)included healthy older adults, while eight studies (21, 29–32, 37, 39, 41) focused on older adults with health conditions. Intervention durations ranged from 4 to 28 weeks, with training sessions conducted two to four times per week. Most studies adopted a progressive resistance training protocol, in which band resistance levels were gradually increased in accordance with participants’ improvements in strength.

**Table 1:** Overview of the studies included into meta-analyses.

Study	Participants (EG/CG)	Age(y)		Proportion of female (%)	Duration (week)	Intervention	Outcome
		EG	CG				
Hanphitakphong et al. (19)	15/14	64.6 ± 2.6	65.5 ± 1.8	51.72	4	3 times/week, 45 min/session	CST, TUG, FRT
Seo et al. (21)	12/10	70.3 ± 5.38	72.9 ± 4.75	100	16	3 times/week, 50 min/session	leg extension test, CST, TUG
Herda et al. (22)	24/13	m: 69.1 ± 6.6 f: 65.5 ± 7.1	m: 69.1 ± 6.6 f: 65.5 ± 7.1	NA	6	2 times/week	TUG
Stojanović et al. (20)	86/82	75.7 ± 8.9	74.5 ± 8.2	NA	12	2 times/week, 55–60 min/session	CST, TUG
Flandez et al. (23)	28/30	67.81 ± 5.21	67.75 ± 4.91	NA	20	2 times/week, 60 min/session	leg extension test, TUG
Choi et al. (24)	15/12	75.1 ± 1.4	72.3 ± 1.4	NA	12	3 times/week, 60 min/session	CST, TUG
Motalebi et al. (27)	21/24	70.1 ± 6.5	71.2 ± 6.8	42.22	12	2 times/week, 50 min/session	FRT
Liao et al. (31)	25/21	66.39 ± 4.49	68.42 ± 5.86	100	12	3 times/week, 35–40 min/session	leg extension test, TUG
Kwak et al. (13)	15/15	80.1 ± 4.7	77.4 ± 5.5	63.33	8	3times/week, 30 min/session	TUG, FRT
Martins et al. (34)	20/20	69.1 ± 6.3	66.2 ± 6.6	30.00	8	2times/week	leg extension test
Yu et al. (35)	12/12	65.5 ± 4.5	65.0 ± 3.4	41.67	5	3 times/week	TUG
Fahlman et al.	46/41	75 ± 1	76 ± 2	NA	16	3 times/week	leg extension test, CST,
Ribeiro et al. (36)	24/24	78.44 ± 3.84	79.78 ± 3.90	66.67	6	3 times/week, 15 min/session	TUG, FRT
Rogers et al. (38)	16/6	74.8 ± 8.8	74.7 ± 4.5	100	4	3 times/week, 50 min/session	CST, TUG
Damush et al. (40)	33/29	68 ± 5.58	68 ± 5.58	NA	8	2 times/week, 45 min//session	leg extension test
Oh et al. (30)	19/19	74.9 ± 1.5	73.5 ± 1.2	100	18	2 times/week, 60 min/session	leg extension test
Jette et al. (39)	107/108	75.4 ± 7.4	74.6 ± 6.5	NA	24	3 times/week, 35 min/session	TUG
O'Shea et al. (37)	27/27	66.9 ± 7.0	68.4 ± 9.9	NA	12	3 times/week	TUG
Chupel et al. (32)	16/17	83.5 ± 5.13	82.12 ± 6.41	100	28	2–3 times/week, 20–30 min/session	CST, FRT
Fritz et al. (29)	ETG: 22/20 EBG: 21/20	ETG: 69.2 ± 1.06 EBG: 70.43 ± 0.97	67.2 ± 1.06	100	8	2 times/week	CST, TUG
Gargallo et al. (28)	high:39/23 mod:31/23	high:71.1 ± 5.3 mod:68.74 ± 6.05	70.46 ± 8.1	100	16	2 times/week, 35–40 min/session	CST, TUG
Park et al. (33)	15/15	73.1 ± 3.0	70.9 ± 3.9	0	24	3 times/week, 20–50 min/session	CST, TUG
Rieping et al. (26)	15/13	83.47 ± 4.92	80.85 ± 10.86	100	14	3 times/week, 45 min/session	CST, TUG
Urzi et al. (25)	11/9	84.4 ± 7.7	88.9 ± 5.3	100	12	3 times/week, 35–40 min/session	CST
Lee et al. (12)	10/10	74 ± 4.6	73 ± 6.4	100	8	4 times/week, 40 min/session	CST
Chen et al. (41)	33/33	77.0 ± 5.2	75.3 ± 6.0	66.15	8	3 times/week, 45–60 min/session	CST

EG, elastic band resistance training group; CG, control group; CST, chair sitting test; TUG, time up and go test; FRT, functional reach test.

### 3.2 Assessment of study quality

**Figure 2:**
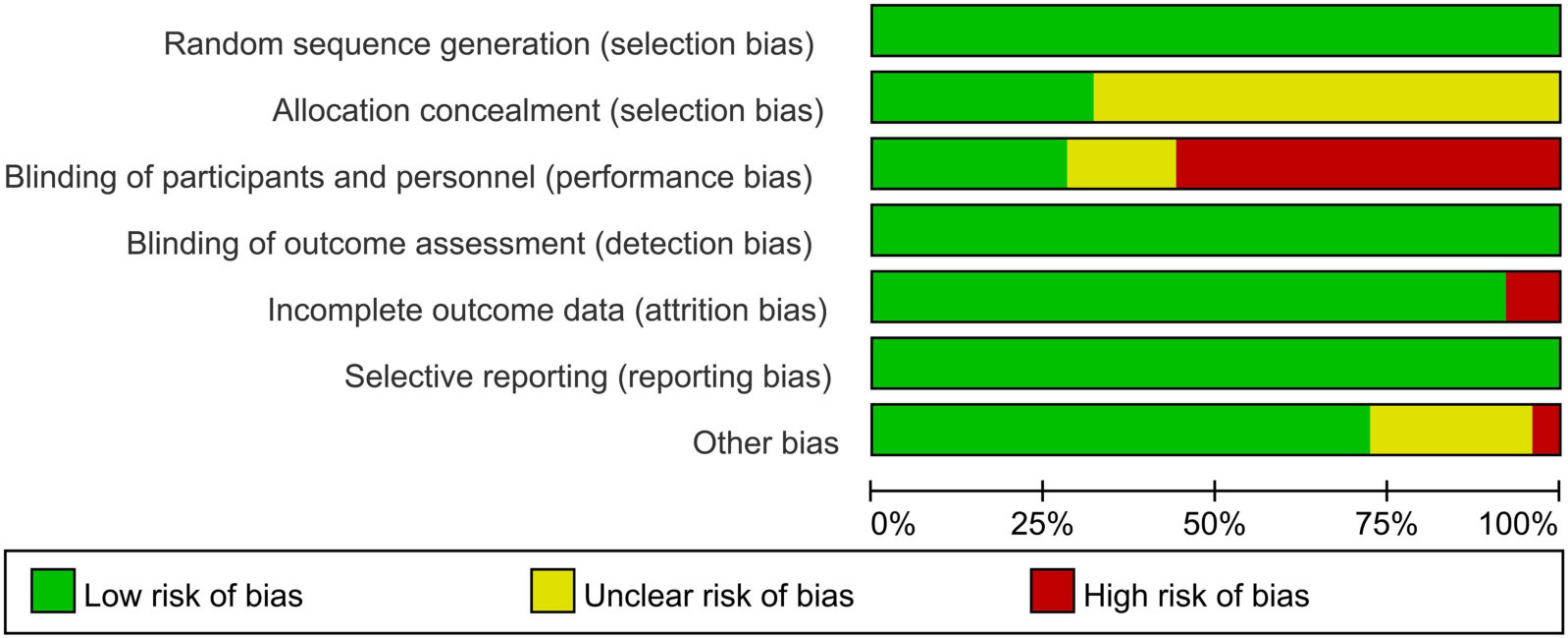
Risk of bias graph of included studies.

**Figure 3:**
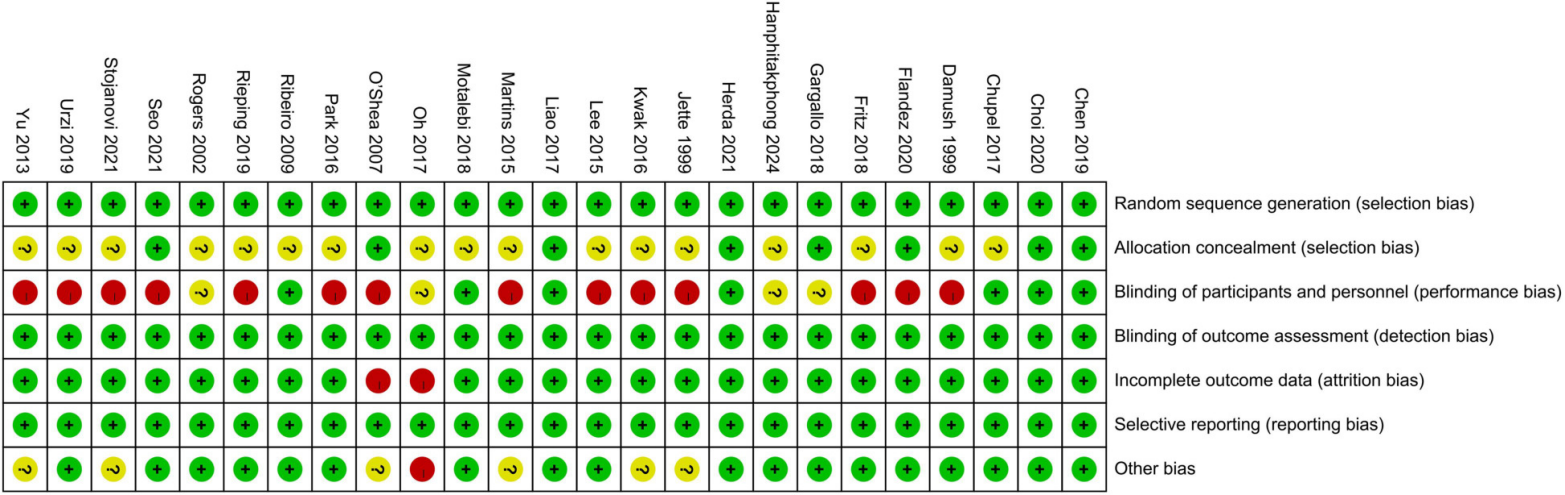
Risk of bias summary of included studies.

Eight studies (21–24, 28, 31, 37, 41) provided a detailed description of their randomization methods. Fourteen studies (12, 13, 20, 21, 23, 25, 26, 29, 33–35, 37, 39, 40) implemented single blinding, while seven studies (22, 24, 27, 31, 32, 36, 41) utilized a double-blind design. Four studies (19, 28, 30, 38) did not clearly report whether double blinding was employed (Figures 2, 3).

### 3.3 Lower limb strength

#### 3.3.1 The influence of elastic band resistance training on leg extension test

**Figure 4:**
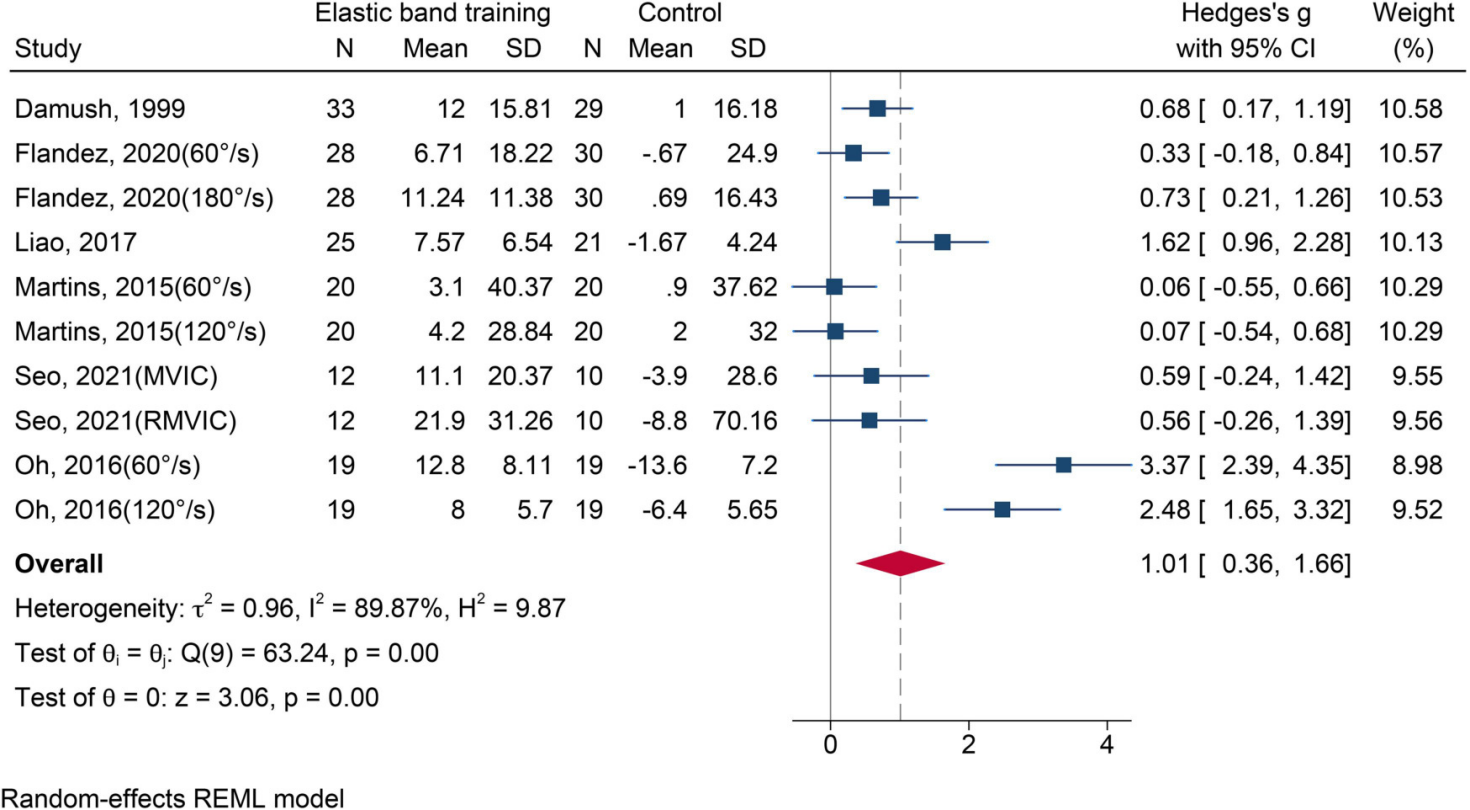
The forest plot of leg extension test between two groups.

Six studies (21, 23, 30, 31, 34, 40) involving 10 effect sizes and a total of 424 participants were included in this analysis. The pooled results revealed a high level of heterogeneity across studies (*I*^2^ = 89.87%). Meta-analysis demonstrated that, compared with the control group, EBT significantly improved leg extension strength [SMD = 1.01, 95% CI (0.36, 1.66), *p* = 0.002] (Figure 4). Funnel plot asymmetry and Egger's test (*p* < 0.05) suggested potential publication bias. After applying a correction, the adjusted effect size remained significant [SMD = 1.12, 95% CI (0.50, 1.75)].

The results of subgroup analysis are shown in Table 2. Gender: Significant improvements were observed in both female [SMD = 1.52, 95% CI (0.61, 2.43), *p* = 0.001] and mixed-gender groups [SMD = 0.33, 95% CI (0.01, 0.64), *p* = 0.043]. Health Status: Both healthy [SMD = 0.40, 95% CI (0.13, 0.68), *p* = 0.004] and non-healthy group [SMD = 1.71, 95% CI (0.66, 2.75), *p* = 0.001] showed significant improvements. Intervention Duration: Interventions lasting 9–12 weeks [SMD = 2.44, 95% CI (1.45, 3.43), *p* < 0.001] and 13–28 weeks [SMD = 0.54, 95% CI (0.23, 0.85), *p* < 0.001] both produced significant effects.

**Table 2:** Results of subgroup analysis.

Subgroup	Study	Participants	Heterogeneity		Meta-analysis results	
			*I*^2^(%)	*P*	SMD (95%CI)	*P*
Leg extension test						
Gender						
Female	4	228	88.87	0.000	1.52 (0.61–2.43)	0.001
Mix gender	2	196	20.72	0.292	0.33 (0.01–0.64)	0.043
Health status						
Healthy	3	258	20.75	0.275	0.40 (0.13–0.68)	0.004
Non-healthy	3	166	87.83	0.000	1.71 (0.66–2.75)	0.001
Duration (week)						
4–8	2	142	40.74	0.191	0.30 (−0.13–0.73)	0.173
9–12	2	122	77.28	0.012	2.44 (1.45–3.43)	0.000
13–28	2	160	0.00	0.761	0.54 (0.23–0.85)	0.001
Chair stand test						
Gender						
male	1	30	n/a	n/a	0.62 (−0.09–1.33)	n/a
female	8	350	98.52	0.000	2.47 (0.35–4.60)	0.023
Mix gender	4	290	93.47	0.000	1.33 (0.16–2.51)	0.026
Health status						
healthy	9	466	77.20	0.000	0.78 (0.35–1.21)	0.000
non-healthy	4	204	98.71	0.000	5.03 (1.03–9.02)	0.014
Duration (week)						
4–8	5	242	98.55	0.000	4.35 (0.84–7.87)	0.015
9–12	3	215	76.89	0.022	0.29 (−0.41 to 0.99)	0.423
13–28	5	219	32.92	0.156	1.08 (0.73–1.43)	0.000
Timed up and go test						
Gender						
Male	7	340	n/a	n/a	−0.24 (−0.93 to 0.46)	n/a
Female	1	30	97.48	0.000	−1.90 (−3.49 to −0.31)	0.019
Mix gender	10	690	98.00	0.000	−1.11 (−2.29 to −0.08)	0.068
Health status						
Healthy	12	607	96.05	0.000	−0.93 (−1.81 to −0.05)	0.038
Non-healthy	6	453	98.58	0.000	−2.31 (−4.31 to −0.30)	0.024
Duration (week)						
4–8	7	273	97.77	0.000	−2.73 (−4.92 to −0.55)	0.014
9–12	4	295	0	0.428	−0.31 (−0.53 to −0.08)	0.008
13–28	7	492	0	0.176	−0.68 (−0.86 to −0.50)	0.000

#### 3.3.2 The influence of elastic band resistance training on chair stand test

**Figure 5:**
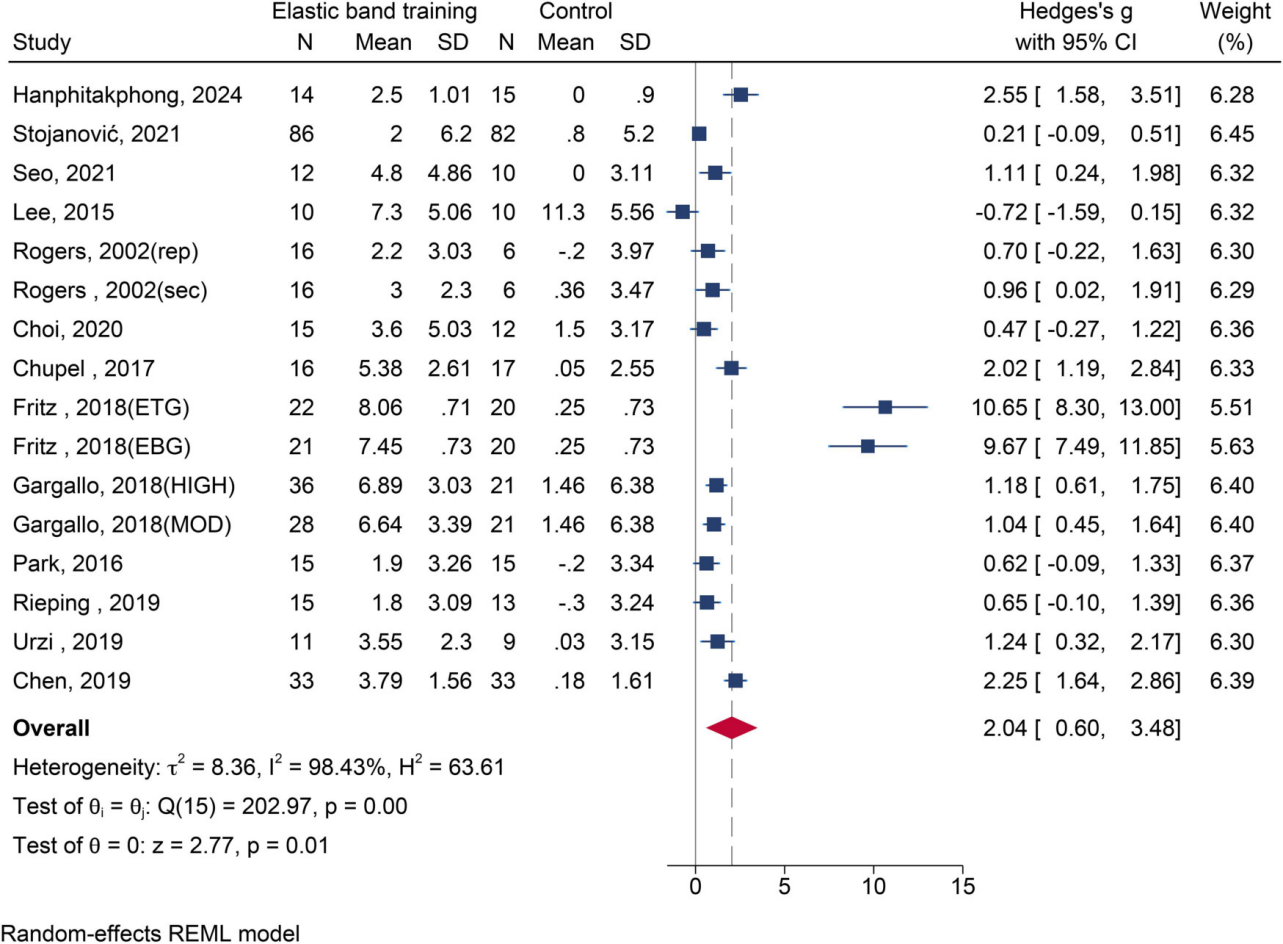
The forest plot of chair stand test between two groups.

Thirteen studies (12, 19–21, 24–26, 28, 29, 32, 33, 38, 41) reporting 16 effect sizes with 670 participants were included. The heterogeneity among these studies was substantial (*I*^2^ = 98.43%). Meta-analysis indicated a statistically significant improvement in chair stand performance following EBT [SMD = 2.04, 95% CI (0.60, 3.48), *p* = 0.006] (Figure 5). Eggers’ test (*p* < 0.05) revealed potential publication bias. After adjustment, the effect remained significant and was even greater [SMD = 2.89, 95% CI (1.60, 4.19)].

The results of subgroup analysis are shown in Table 2. Gender: Significant effects were seen in both female [SMD = 2.47, 95% CI (0.35, 4.60), *p* = 0.023] and mixed-gender groups [SMD = 1.33, 95% CI (0.16, 2.51), *p* = 0.026].Health Status: Improvements were evident in both healthy [SMD = 0.78, 95% CI (0.35, 1.21), *p* < 0.001] and non-healthy group [SMD = 5.03, 95% CI (1.03, 9.02), *p* = 0.014]. Intervention Duration: Interventions lasting 4∼8 weeks [SMD = 4.35, 95% CI (0.84, 7.87), *p* = 0.015] and 13∼28 weeks [SMD = 1.08, 95% CI (0.73, 1.43), *p* < 0.001] both produced significant effects.

### 3.4 Balance function

#### 3.4.1 The influence of elastic band resistance training on timed up and go test

**Figure 6:**
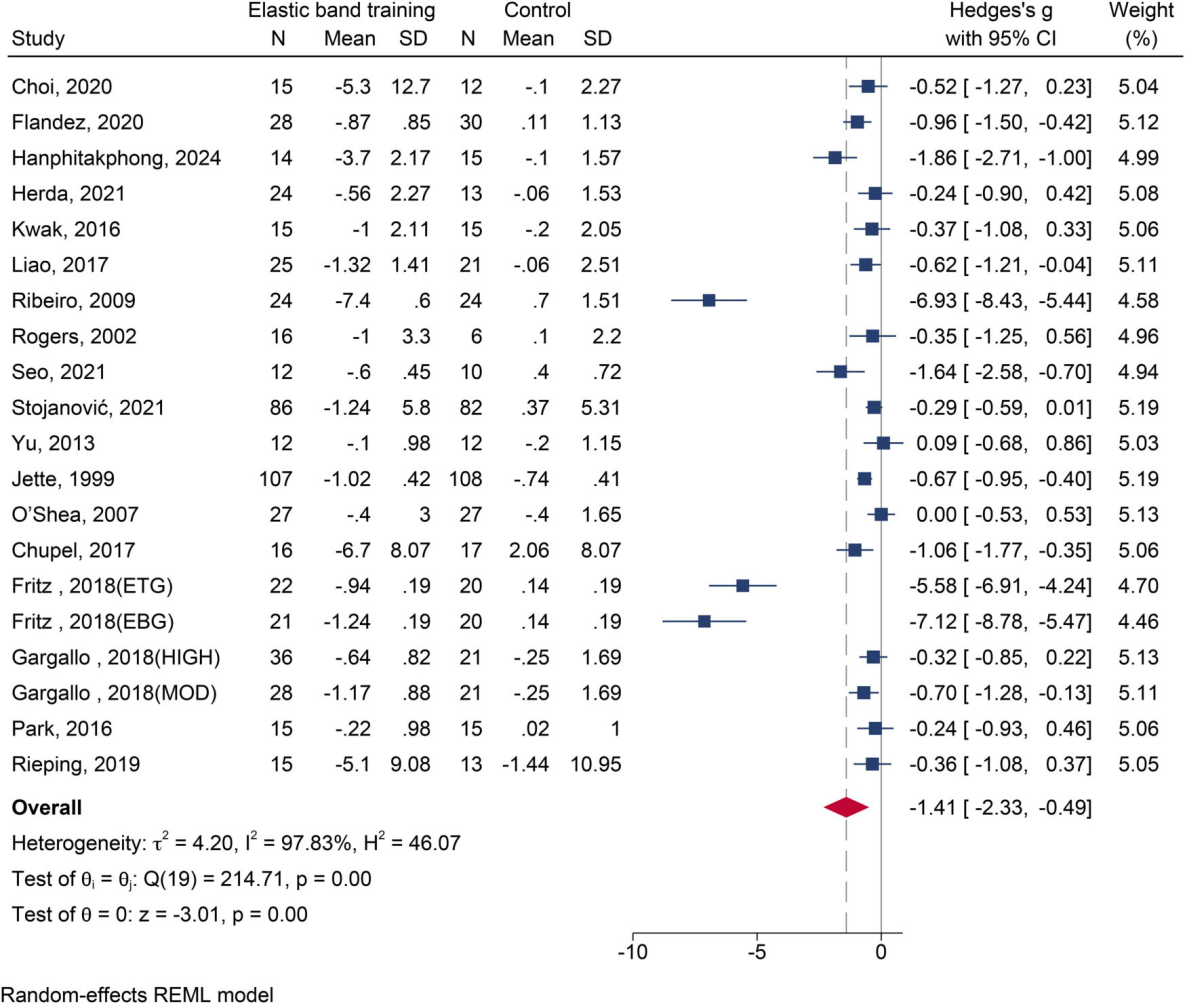
The forest plot of timed up and go test between two groups.

Eighteen studies (13, 19–24, 26, 28, 29, 31–33, 35–39) with 20 effect sizes and 1,060 participants were included. A high degree of heterogeneity was observed (*I*^2^ = 97.83%). The meta-analysis showed that EBT significantly improved performance on the TUG test compared to the control group [SMD = –1.41, 95% CI (–2.33, −0.49), *p* = 0.003] (Figure 6). Publication bias was detected (Egger's test, *p* < 0.05), and the corrected effect size remained significant [SMD = –1.85, 95% CI (–2.66, −1.04)].

The results of subgroup analysis are shown in Table 2. Gender: A significant effect was found in female participants [SMD = –1.90, 95% CI (–3.49, −0.31), *p* = 0.019]. Health Status: Significant improvements were observed in both healthy [SMD = –0.93, 95% CI (–1.81, −0.05), *p* = 0.038] and non-healthy groups [SMD = –2.31, 95% CI (–4.31, −0.30), *p* = 0.024]. Intervention Duration: All durations showed statistically significant improvements.

#### 3.4.2 The influence of elastic band resistance training on functional reach test

**Figure 7:**
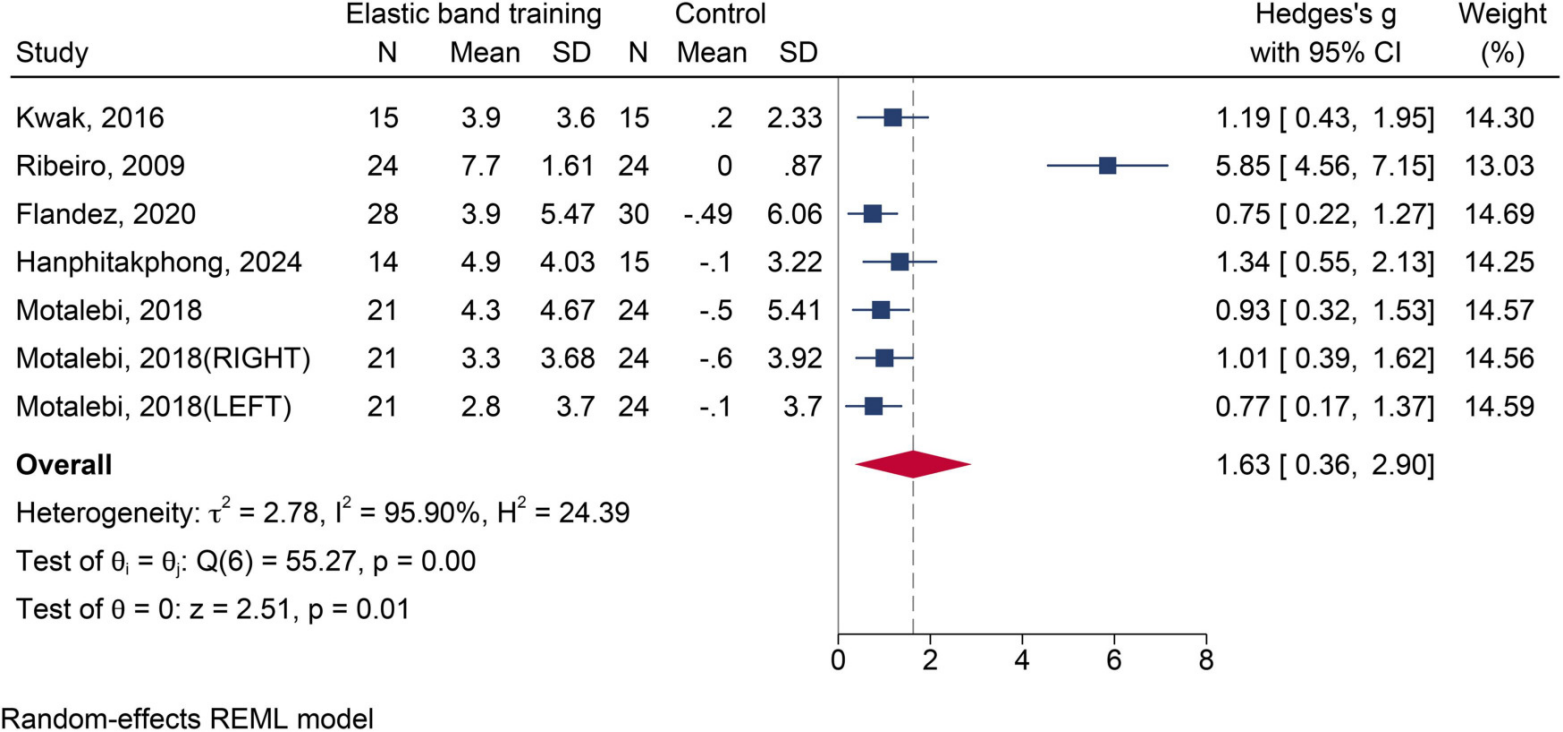
The forest plot of functional reach test between two groups.

Five studies (13, 19, 23, 27, 36) with seven effect sizes and a total of 300 participants were included. Substantial heterogeneity was identified (*I*^2^ = 95.90%). Meta-analysis revealed that EBT significantly improved scores on the FRT compared with the control group [SMD = 1.63, 95% CI (0.36, 2.90), *p* = 0.012] (Figure 7). Egger's test (*p* < 0.05) suggested the presence of publication bias. After correction, the adjusted effect size remained statistically significant [SMD = 1.99, 95% CI (0.94, 3.07)].

## 4 Discussion

This study suggests that EBT may improve lower limb muscle strength and balance in older adults. Based on the synthesis of 25 RCTs, the findings indicate that EBT could be an effective intervention across various populations and training contexts. The results also suggest that intervention duration might influence outcomes: increases in muscle strength appeared more consistent in programs lasting over 8 weeks, while balance improvements were observed even with shorter durations, potentially due to neural adaptations. Subgroup analyses indicated that older adults with varying health conditions may benefit from EBT. The more pronounced intervention effects observed in women may be partly attributable to the greater number of studies conducted with female participants. Overall, these findings support the potential of EBT as a feasible and adaptable exercise option for geriatric populations, although further high-quality studies are needed to confirm these effects.

### 4.1 The effect of elastic bands resistance training on lower limb strength

Compared to the control group, EBT significantly improved lower limb muscle strength in older adults. According to the results of the leg extension test and the CST, training durations exceeding 12 weeks yield the most favorable outcomes in terms of improving lower limb extensor strength. This finding aligns with the meta-analysis by Martins et al. (15), which reported that progressive elastic resistance training has a strong positive effect on muscle strength in both healthy and functionally impaired older adults, and a moderate effect in those with clinical conditions. However, Martins et al. (15) included a relatively small number of studies and focused exclusively on muscle strength. In contrast, the present study assessed both lower limb strength and balance function and further examined potential sources of heterogeneity through subgroup analyses.

Decreased leg extension strength has been recognized as a major cause of decreased mobility and increased risk of falls in older adults (42). Our findings suggest that EBT is effective in improving leg extension in this population. It is important to note that this analysis included only one study with a male-only sample; therefore, the specific effects of EBT on leg extension strength in older men remain to be clarified. Subgroup analyses based on health status indicated that EBT had a significant and consistent effect on healthy older adults. Although significant effects were also observed in participants with health problems, there was substantial heterogeneity, which may be due to the inclusion of individuals with different conditions, such as functional limitations, metabolic syndrome, sarcopenia, and chronic obstructive pulmonary disease.

The CST is widely used as a functional measure of lower limb strength in older adults (15). *n* this meta-analysis, EBT significantly increased CST performance, consistent with the findings of Oliveira (16)and Martinez (14). Differences in chair characteristics and testing procedures across studies may have contributed to this variation. Upon reviewing the included trials, methodological inconsistencies were noted in parameters such as chair height, knee flexion angle during sitting, and hand placement during testing. Some studies failed to report these details entirely. Shamay et al. (43) highlighted that chair height is a key determinant of CST performance: lower chairs increase torque demands and range of motion at the hip and knee, making the test more challenging, whereas higher chairs reduce the workload on the lower limbs and the need for knee extensor stabilization.

### 4.2 The effect of elastic bands resistance training on balance function

This meta-analysis demonstrated that EBT significantly improved balance function in older adults compared to control groups. This finding is consistent with the meta-analysis by Martinez et al. (14), although their analysis included fewer studies and focused solely on healthy participants. In contrast, the present study incorporated a broader population, including individuals with varying health conditions, thereby offering more generalizable evidence. Impaired balance is widely recognized as a leading risk factor for falls in older adults (44). It often manifests as instability during postural tasks such as standing, weight shifting, walking, reacting to external perturbations, and performing transitions. Enhancing balance is thus essential for improving postural control and maintaining functional independence. Structured exercise interventions, including EBT, have consistently been shown to improve balance and reduce fall risk in aging populations (3, 45).

The TUG is a widely used, practical measure of functional mobility in older adults, particularly those with frailty. Its popularity stems from its low cost, ease of use, and strong correlation with more complex assessments of gait speed, balance, and overall functional status (46, 47). Poor performance on the TUG test is not only linked to an increased risk of falls but is also associated with cognitive decline and higher mortality (46, 47). Evidence suggests that each one-second reduction in TUG time corresponds to an approximate 9% decrease in fall risk (48). This study found that EBT significantly reduced the time to TUG completion. Subgroup analyses showed significant improvements in all intervention durations, with particularly low heterogeneity observed for durations longer than 8 weeks. However, not all included studies reported significant changes. For instance, Yu (35) and Shea (37) found no notable improvements. Yu et al. (35) attributed this to the low intensity and poor specificity of their intervention, which consisted mainly of light stretching over a short duration—likely insufficient to elicit neuromuscular adaptations. Shea et al. (37) noted that low adherence may have diluted the observed effects. Subgroup analysis based on health status showed that EBT benefited both healthy and medically compromised older adults, supporting its broad applicability. However, beyond the efficacy of EBT, participant adherence and physical capacity to complete training programs must also be considered—especially in clinical populations (37). Overall, progressive EBT programs lasting longer than eight weeks appear particularly effective for promoting neuromuscular adaptations and improving balance.

The FRT is another widely used clinical assessment of balance. It is valued for its portability, affordability, reliability, and sensitivity, making it ideal for identifying balance impairments and tracking changes in postural stability over time (49). This meta-analysis found that EBT significantly improved FRT scores. Notably, Ribeiro et al. (36) observed large effect sizes in both TUG and FRT scores after a program that focused solely on ankle-strengthening exercises. These results suggest that enhancing plantar flexor strength is closely linked to improvements in balance performance. Ankle musculature—including both dorsiflexors and plantar flexors—plays a critical role in maintaining postural stability. This is especially true for older adults with a history of falls, for whom muscle weakness is strongly associated with balance dysfunction (50). For example, dorsiflexors help stabilize the body during backward sway or slips, while plantar flexors are essential for preventing forward displacement of the center of mass beyond the base of support (50).

Subgroup analyses also showed that interventions lasting less than 8 weeks did not significantly improve leg extension strength, whereas interventions lasting more than 12 weeks significantly improved leg extension strength with reduced heterogeneity. While initial strength improvements are often driven by neural mechanisms, meaningful increases typically require hypertrophy and expansion of muscle cross-sectional area. In older adults, this process is slower due to decreased anabolic capacity and motor unit loss. Additionally, the relatively low external load provided by resistance bands and the use of progressive protocols may lead to delayed hypertrophic responses unless accumulated training volume is sufficient. In contrast, balance function improved significantly even with shorter interventions, and heterogeneity was notably lower in programs lasting 8 weeks or more. Balance control relies on the integration of proprioceptive, vestibular, and visual inputs, as well as neuromuscular coordination. Improvements in these systems—such as increased motor unit recruitment, improved intermuscular coordination, and enhanced postural feedback—occur relatively quickly through neural adaptation, particularly during the early stages of training. Although resistance bands provide low mechanical load, the continuous adjustments in posture and movement they demand effectively stimulate neuromuscular control. In conclusion, for improving lower limb strength, EBT programs should last at least 8 weeks. For balance improvements, effective outcomes can be achieved with durations of 4 weeks or more.

### 4.3 Limitations

This study systematically synthesized current evidence based on strict adherence to predefined inclusion and exclusion criteria and established review methodology, and suggested potential positive effects of EBT on improving lower extremity strength and balance function in older adults. However, several limitations should be considered: (a) Methodological limitations: The quality of the included RCTs was variable. In particular, elements such as blinding and allocation concealment were often unclearly reported, and many studies had relatively small sample sizes. These factors may introduce bias and compromise the overall reliability of the findings. (b) Heterogeneity of intervention protocols: The included studies exhibited considerable variability in training parameters, which led to substantial heterogeneity in the results and limited the development of an optimal intervention protocol. (c) Limited data for specific outcomes: Due to the limited number of studies targeting certain subgroups, further subgroup analyses were constrained. (d) Absence of long-term follow-up: Most studies focused on short-term effects, with limited data on the long-term sustainability of the observed improvements or their impact on fall incidence over time.

## 5 Conclusion

In summary, elastic band resistance training appears to be a feasible intervention for improving lower limb strength and balance function in older adults with different health conditions. Strength gains seem more evident with interventions lasting at least 8 weeks, while balance improvements may occur after 4 weeks or more. It is recommended that the training duration be extended to 12 weeks or longer. The results of this study indicate that such a training period contributes to achieving significant and stable improvements in lower limb strength and balance function. Given its affordability, safety, and adaptability, EBT can be recommended as a valuable strategy in fall prevention and mobility enhancement programs for the aging population.

However, the methodological quality of the included studies varies substantially, and considerable heterogeneity exists across intervention protocols. To further validate these findings and optimize their clinical application, future research should prioritize the design and implementation of high-quality, large-scale, multicenter RCTs with rigorous methodological standards and standardized intervention procedures. In addition, greater standardization in the selection and reporting of outcome measures is needed to reduce sources of heterogeneity and enhance comparability across studies. The majority of studies included in this paper primarily focused on short-term effects, with a scarcity of long-term follow-up data. Articles investigating long-term outcomes are relatively limited, necessitating further validation in subsequent research.

## Data Availability

The original contributions presented in the study are included in the article/Supplementary Material, further inquiries can be directed to the corresponding authors.
